# Non-HDL Cholesterol as a Practical Gatekeeper for Adolescent Dyslipidemia Screening: Implications for Nutrition and Public Health

**DOI:** 10.3390/nu18142285

**Published:** 2026-07-13

**Authors:** Kazufumi Nakamura, Taiji Okada, Nobuhide Watanabe, Hirotomo Sato, Yuzo Kagawa, Hiroshi Kawahara, Takahiro Sakamoto, Seita Yamasaki, Junya Tanabe, Madoka Furuta, Yuka Kawanami, Yuko Miki, Akihiro Endo

**Affiliations:** Department of Cardiovascular Medicine, Faculty of Medicine, Shimane University, Izumo 693-8501, Japan; okada@med.shimane-u.ac.jp (T.O.); n-wata@med.shimane-u.ac.jp (N.W.); h-sato@med.shimane-u.ac.jp (H.S.); kagawa@med.shimane-u.ac.jp (Y.K.); a0703@med.shimane-u.ac.jp (H.K.); t.saka@med.shimane-u.ac.jp (T.S.); seyama86@med.shimane-u.ac.jp (S.Y.); jtanabe@med.shimane-u.ac.jp (J.T.); mdyamag@med.shimane-u.ac.jp (M.F.); nami-wo@med.shimane-u.ac.jp (Y.K.); mikiyuko@med.shimane-u.ac.jp (Y.M.); akiendo@med.shimane-u.ac.jp (A.E.)

**Keywords:** non-HDL cholesterol, adolescent, dyslipidemia

## Abstract

Dyslipidemia in adolescents represents an early and potentially modifiable stage in the lifelong trajectory of cardiovascular disease. However, optimal lipid screening strategies in this age group remain controversial, particularly regarding feasibility, biological relevance, and reliance on family history. Non-high-density lipoprotein cholesterol (non-HDL cholesterol) has emerged as a promising screening marker because it reflects the total burden of atherogenic lipoproteins and can be reliably assessed even without fasting, making it particularly suitable for large-scale pediatric screening settings. A recent nationwide population-based study of Korean adolescents provides robust real-world evidence supporting the utility of non-HDL cholesterol in dyslipidemia screening, across both sexes and independent of familial lipid risk. From a nutritional and public health perspective, non-HDL cholesterol is closely related to dietary habits, postprandial hyperlipidemia, and metabolic stress, and is particularly important in the modern food environment characterized by increased consumption of ultra-processed foods. This Opinion discusses the biological rationale, clinical feasibility, and population-level implications of non-HDL cholesterol-based screening in adolescents. By linking lipid biology with nutrition-focused prevention and lifelong cardiovascular health, non-HDL cholesterol may serve as a practical gatekeeper for early risk identification and upstream intervention.

## 1. Why Adolescent Dyslipidemia Screening Deserves Renewed Attention

Dyslipidemia in childhood and adolescence is one of the earliest detectable stages in the lifelong trajectory of cardiovascular disease [1,2]. Although atherosclerotic cardiovascular disease usually develops decades later, accumulating evidence indicates that adverse lipid profiles, endothelial dysfunction, and early vascular changes begin to manifest, especially during adolescence, in an environment characterized by unhealthy diets, increasing obesity, and insulin resistance [3,4]. Longitudinal cohort studies further demonstrate that lipid-related risk factors present in childhood track into adulthood and are associated with subclinical atherosclerosis and future cardiovascular disease, supporting the importance of early identification of adverse lipid phenotypes [5].

Despite this recognition, optimal strategies for lipid screening in adolescents remain a subject of ongoing debate [2,6]. Traditional approaches have relied on fasting lipid measurements and selective screening based on family history of dyslipidemia or premature cardiovascular disease. However, these strategies face important limitations in real-world settings. Fasting requirements pose logistical challenges, particularly in school-based or community-based health examinations, while family history is often incomplete, inaccurately reported, or unavailable. As a result, a substantial proportion of adolescents with emerging cardiometabolic risk may remain unidentified. These uncertainties are reflected in persistent discrepancies among guideline recommendations regarding universal versus selective lipid screening strategies in youth [3,7].

At the same time, the epidemiological context surrounding adolescent health has changed substantially. Modern dietary environments, characterized by increased consumption of ultra-processed foods, excess saturated fat, and refined carbohydrates, have shifted the dominant drivers of dyslipidemia from purely genetic predisposition toward lifestyle-related and metabolic factors. In this setting, lipid abnormalities may arise even in the absence of a clear familial risk, calling into question the adequacy of selective screening paradigms developed in earlier eras. Contemporary scientific statements on pediatric cardiovascular prevention increasingly emphasize the interaction between dietary exposures, adiposity, insulin resistance, and early lipid abnormalities in shaping long-term cardiovascular risk trajectories [4].

These challenges underscore the need for screening markers that are not only biologically meaningful but also operationally feasible and broadly applicable. An ideal marker for adolescent lipid screening should capture early atherogenic risk, reflect diet-related metabolic stress, and be easily integrated into routine health assessments without imposing excessive burden on individuals or healthcare systems. Importantly, such a marker should facilitate constructive, prevention-oriented dialogue centered on nutrition and lifestyle modification rather than disease labeling.

Non-high-density lipoprotein (non-HDL) cholesterol has emerged as a promising candidate in this regard. By encompassing the total burden of atherogenic lipoproteins, non-HDL cholesterol addresses several of the practical barriers that have historically limited pediatric lipid screening. The recent national population-based study conducted among Korean adolescents provides timely and compelling evidence supporting its utility, offering an opportunity to reconsider how dyslipidemia screening can be optimized in contemporary nutritional and public health contexts [8].

This Opinion article builds on these findings to discuss the broader implications of non-HDL cholesterol-based screening from a nutritional, clinical, and life-course prevention perspective. By examining how non-HDL cholesterol aligns with modern dietary exposures, postprandial lipid metabolism, and long-term cardiovascular risk, this article argues that non-HDL cholesterol may serve as a practical gatekeeper for adolescent dyslipidemia screening in real-world settings [9]. Nevertheless, direct evidence demonstrating that non-HDL cholesterol-guided screening and intervention during adolescence ultimately improve adult cardiovascular outcomes remains limited. Therefore, the present discussion should be interpreted as supporting non-HDL cholesterol as a practical and biologically plausible tool for early risk identification rather than as evidence that its use improves long-term cardiovascular outcomes.

## 2. Non-HDL Cholesterol: A Biologically Sound and Clinically Feasible Marker

Non-HDL cholesterol, calculated as total cholesterol minus HDL cholesterol, represents the cholesterol content of all atherogenic apolipoprotein B-containing lipoproteins, including low-density lipoprotein (LDL), very-low-density lipoprotein (VLDL), intermediate-density lipoprotein (IDL), lipoprotein (a), and remnant particles [10]. From a pathophysiological standpoint, non-HDL cholesterol therefore reflects the total atherogenic lipoprotein burden more comprehensively than LDL cholesterol alone, particularly in states characterized by insulin resistance, obesity, and postprandial dyslipidemia-conditions that are increasingly prevalent among adolescents worldwide.

A major practical advantage of non-HDL cholesterol is that it does not require fasting blood samples. This characteristic is particularly relevant in pediatric and adolescent populations, in whom fasting compliance is often suboptimal and may limit the feasibility of large-scale or school-based screening programs. In real-world clinical and public health settings, fasting requirements have historically represented a substantial barrier to widespread lipid screening, leading to missed opportunities for early risk identification. Non-HDL cholesterol can be reliably assessed even in non-fasting conditions while maintaining strong biological relevance [11].

Although direct measurement of LDL cholesterol is feasible in non-fasting conditions, calculated LDL cholesterol may be affected by postprandial increases in triglyceride levels. Because non-HDL cholesterol does not rely on triglyceride-based estimation formulas, it may provide a more practical and robust metric in routine screening settings.

Several clinical guidelines have acknowledged the utility of non-HDL cholesterol in pediatric populations. The National Heart, Lung, and Blood Institute (NHLBI) and the American Academy of Pediatrics (AAP) have recognized non-HDL cholesterol as an acceptable and, in some contexts, preferred screening marker for dyslipidemia in children and adolescents [3]. More recently, European guidelines have also emphasized the importance of apolipoprotein B-containing lipoproteins and non-HDL cholesterol in cardiovascular risk assessment [12].

It should be emphasized that LDL cholesterol remains the primary therapeutic target in pediatric dyslipidemia, particularly in familial hypercholesterolemia, where early identification and intervention are essential for long-term cardiovascular risk reduction [13]. In this context, non-HDL cholesterol should not be viewed as a replacement for LDL cholesterol. Rather, it provides complementary information by capturing the broader burden of atherogenic apolipoprotein B–containing lipoproteins, including triglyceride-rich remnant particles that are not fully reflected by LDL cholesterol alone. Accordingly, non-HDL cholesterol may be particularly valuable as a first-line screening marker, whereas LDL cholesterol remains central to diagnostic confirmation and therapeutic decision-making.

It should also be recognized that non-HDL cholesterol has certain limitations. Because it reflects the cholesterol content rather than the number of atherogenic particles, discordance between non-HDL cholesterol and apolipoprotein B may occur, particularly in individuals with obesity, insulin resistance, or hypertriglyceridemia. In such circumstances, apolipoprotein B may provide additional information regarding atherogenic particle number and cardiovascular risk [14]. Therefore, non-HDL cholesterol and apolipoprotein B should be viewed as complementary rather than competing biomarkers, with non-HDL cholesterol offering a simple and widely accessible screening tool and apolipoprotein B providing further refinement of cardiovascular risk assessment when clinically indicated.

Despite these endorsements, uncertainty remains regarding the real-world diagnostic performance of non-HDL cholesterol at the population level, particularly across different ethnic and dietary backgrounds.

From a nutritional perspective, non-HDL cholesterol is especially attractive because it is closely linked to dietary patterns and metabolic health. Diets high in saturated fat, refined carbohydrates, and ultra-processed foods are associated with elevated levels of triglyceride-rich lipoproteins and remnant cholesterol, which are captured by non-HDL cholesterol but may be underestimated when focusing solely on LDL cholesterol. Conversely, improvements in diet quality—such as increased intake of fiber, unsaturated fats, and whole foods—are reflected in favorable changes in non-HDL cholesterol, making it a potentially useful marker for monitoring the effectiveness of nutritional interventions in adolescents [4,15].

Importantly, non-HDL cholesterol combines simplicity with robustness. While it is mathematically straightforward, it integrates complex lipoprotein metabolism into a single clinically interpretable value. This balance between biological depth and clinical practicality positions non-HDL cholesterol as a “simple but not simplistic” metric. In the context of adolescent health, where screening strategies must balance accuracy, feasibility, and acceptability, non-HDL cholesterol emerges as a compelling candidate for a first-line screening marker.

Against this background, population-based evaluations of non-HDL cholesterol are essential to determine whether its theoretical advantages translate into consistent diagnostic performance in real-world settings. The recent national study conducted in Korean adolescents provides important evidence in this regard, offering insights that extend beyond traditional family history-based screening paradigms and informing future nutrition-focused prevention strategies [8].

## 3. What the Korean National Population-Based Study Adds

The recent national population-based study conducted among Korean adolescents provides important and timely evidence supporting the role of non-HDL cholesterol in pediatric dyslipidemia screening. Using data from the Korean National Health and Nutrition Examination Survey, the investigators analyzed a large, nationally representative cohort of adolescents aged 10–19 years, allowing for robust estimation of lipid abnormalities at the population level [8]. Such large-scale evaluations are particularly valuable in pediatric research, where evidence is often derived from smaller or clinic-based samples.

One of the key contributions of this study lies in its demonstration of the high specificity of non-HDL cholesterol for identifying elevated LDL cholesterol in both boys and girls. A non-HDL cholesterol threshold of 145 mg/dL detected directly measured or calculated LDL cholesterol abnormalities with specificity exceeding 99% across sexes. This finding is clinically meaningful, as high specificity is especially desirable in screening contexts to minimize false-positive results and unnecessary follow-up testing. In adolescent populations, where overdiagnosis may carry psychological and social consequences, a screening marker with strong rule-in capability is of particular importance.

In addition to its high specificity, non-HDL cholesterol demonstrated moderate-to-high sensitivity for detecting elevated LDL cholesterol, with performance that was comparable to or better than that of total cholesterol. Notably, non-HDL cholesterol consistently outperformed total cholesterol in identifying adolescents with LDL cholesterol abnormalities. This observation supports the concept that non-HDL cholesterol provides incremental diagnostic value beyond traditional lipid measures, even when derived from the same lipid panel.

A further strength of this study is its examination of familial lipid risk. Traditionally, pediatric lipid screening strategies have relied heavily on family history of dyslipidemia or premature cardiovascular disease to guide selective testing. However, the Korean study demonstrated that the association between elevated non-HDL cholesterol and dyslipidemia remained stable regardless of parental lipid status. Although a positive family history was associated with a higher overall risk of dyslipidemia, non-HDL cholesterol maintained its discriminatory ability independent of familial factors. This finding challenges the long-standing paradigm of family history-based screening and supports the use of non-HDL cholesterol as a more universally applicable marker.

From a public health perspective, the use of nationally representative data strengthens the generalizability of these findings. Korea has undergone rapid nutritional and lifestyle transitions, characterized by increased consumption of energy-dense and ultra-processed foods alongside rising rates of adolescent obesity. In such environments, lipid abnormalities may develop even without a clear familial predisposition. The ability of non-HDL cholesterol to capture dyslipidemia under these conditions underscores its relevance in contemporary, nutrition-driven cardiometabolic risk environments.

Importantly, this study moves the field beyond theoretical or guideline-based endorsement of non-HDL cholesterol by providing real-world, population-level performance data. By demonstrating that non-HDL cholesterol reliably identifies adolescents with dyslipidemia across diverse risk profiles, the findings offer practical reassurance for clinicians and policymakers considering its broader implementation. In the context of adolescent health screening, where feasibility, scalability, and accuracy must be balanced, the Korean national study provides compelling population-level evidence supporting the clinical utility of non-HDL cholesterol.

Nevertheless, caution is warranted when extrapolating these findings to other populations. Genetic backgrounds, dietary patterns, obesity prevalence, and cardiometabolic risk profiles vary substantially across countries and may influence both the distribution of non-HDL cholesterol and its relationship with other lipid parameters. For example, populations with higher rates of obesity and insulin resistance may exhibit greater contributions of triglyceride-rich lipoproteins and remnant cholesterol to overall atherogenic burden. Therefore, additional population-based studies in diverse ethnic and nutritional settings are needed to determine the generalizability of the Korean findings and to refine context-specific screening strategies. Importantly, however, the findings of the Korean study are broadly consistent with international guideline recommendations, which recognize non-HDL cholesterol as a practical marker of atherogenic lipoprotein burden and an acceptable screening tool for dyslipidemia in children and adolescents [3,12]. Nevertheless, direct evidence demonstrating that non-HDL cholesterol-guided screening and intervention during adolescence ultimately improve adult cardiovascular outcomes remains limited [16].

## 4. Beyond Family History: Rethinking Pediatric Lipid Screening Strategies

Family history of dyslipidemia or premature cardiovascular disease has long served as a cornerstone of selective lipid screening strategies in children and adolescents [16]. This approach was originally designed to identify individuals with inherited lipid disorders, such as familial hypercholesterolemia, while limiting unnecessary testing in low-risk populations. However, the epidemiological and nutritional landscape surrounding adolescent health has changed substantially over recent decades [17], raising important questions about the adequacy of family history-based screening in contemporary settings.

One fundamental limitation of relying on family history is its incomplete sensitivity [18,19]. Parental dyslipidemia may remain undiagnosed, particularly in younger parents or in health systems with limited routine screening. In addition, family history is often self-reported and subject to recall bias, which may further reduce its reliability. As a result, a substantial proportion of adolescents with lipid abnormalities may be overlooked when screening is restricted to those with an apparent familial risk.

Importantly, the limitations of family history-based screening should not be interpreted as evidence against the biological importance of genetic determinants of dyslipidemia. Family history represents an imperfect surrogate for inherited susceptibility and may fail to capture the complex genetic architecture underlying lipid metabolism. Numerous studies have demonstrated significant associations between genetic polymorphisms and atherogenic lipid traits, including non-HDL cholesterol, LDL cholesterol, and apolipoprotein B concentrations [20,21,22]. Therefore, contemporary dyslipidemia should be viewed as the result of interactions between genetic predisposition and environmental exposures rather than as a simple dichotomy between inherited and lifestyle-related factors.

More importantly, the dominant drivers of dyslipidemia in today’s adolescents increasingly extend beyond genetic predisposition [23,24,25]. Dietary patterns characterized by high intake of saturated fats, refined carbohydrates, and ultra-processed foods, combined with sedentary behavior and rising obesity rates, have created an environment in which dyslipidemia can develop independently of family history. In such contexts, lipid abnormalities may reflect shared environmental exposures rather than inherited traits, rendering family history an insufficient proxy for risk.

The Korean national study directly addresses this issue by demonstrating that non-HDL cholesterol retains its discriminatory power regardless of parental lipid status [8]. Although adolescents with a positive family history showed a higher overall prevalence of dyslipidemia, the performance of non-HDL cholesterol as a screening marker remained stable across familial risk strata. This finding suggests that non-HDL cholesterol captures risk that is not fully explained by heredity alone and supports its use in broader, more inclusive screening strategies.

From a public health standpoint, these observations argue in favor of shifting from selective, family history-based screening toward more universal or opportunistic approaches. Non-HDL cholesterol is particularly well suited to this paradigm because it can be measured without fasting, incorporated into routine health examinations, and interpreted consistently across diverse risk profiles. By lowering logistical barriers and reducing dependence on accurate family history, non-HDL cholesterol enables more equitable identification of adolescents at cardiometabolic risk.

Importantly, moving beyond family history does not imply disregarding genetic risk altogether. Rather, non-HDL cholesterol may serve as an initial gatekeeper, identifying adolescents who warrant further evaluation, including detailed family assessment, genetic consideration, or targeted intervention. In this sense, non-HDL cholesterol complements rather than replaces traditional risk stratification tools.

Taken together, the evidence supports a reappraisal of pediatric lipid screening strategies in the modern nutritional environment. As lifestyle-related dyslipidemia becomes increasingly prevalent among adolescents, screening frameworks must adapt accordingly. By providing a family history-independent, biologically meaningful, and operationally feasible measure of atherogenic lipoprotein burden, non-HDL cholesterol offers a pragmatic solution for bridging the gap between evolving risk profiles and effective early prevention.

## 5. Nutrition-Focused Prevention and Public Health Implications

The growing recognition of non-HDL cholesterol as a useful screening marker in adolescents has important implications for nutrition-focused prevention strategies and public health policy. Non-HDL cholesterol encompasses all atherogenic apolipoprotein B-containing lipoproteins and can be reliably assessed in the non-fasting state, with only minimal differences between fasting and non-fasting measurements [26]. This characteristic aligns well with contemporary recommendations that routine lipid profiles, including LDL-C and non-HDL-C, need not be restricted to the fasting state.

Importantly, the potential advantages of non-HDL cholesterol discussed in this article primarily relate to its role as a practical screening tool and should not be interpreted as diminishing the central importance of LDL cholesterol in the diagnosis and management of familial hypercholesterolemia and other monogenic lipid disorders.

This feature is particularly relevant in the context of modern dietary patterns, where repeated postprandial lipid excursions and sustained exposure to triglyceride-rich and remnant lipoproteins, rather than isolated fasting abnormalities, may contribute to early vascular injury. By capturing the cumulative burden of atherogenic lipoproteins across both fasting and postprandial states, non-HDL cholesterol provides a practical and biologically meaningful target for nutrition-focused interventions and population-level screening in adolescents.

Postprandial hyperlipidemia is increasingly recognized as a key metabolic disturbance linking diet to atherosclerotic risk [27,28]. After typical meals, especially those rich in saturated fat and refined carbohydrates, triglyceride-rich lipoproteins and their remnants circulate for prolonged periods, promoting endothelial dysfunction, oxidative stress, and low-grade inflammation [27,28,29]. Importantly, these postprandial abnormalities may be present even when fasting LDL cholesterol levels remain within the normal range. In adolescents with obesity or insulin resistance, such exaggerated postprandial responses are often among the earliest manifestations of dyslipidemia [30].

Non-HDL cholesterol inherently incorporates cholesterol carried by very-low-density lipoproteins, intermediate-density lipoproteins, and remnant particles, thereby providing an integrated estimate of both fasting and postprandial atherogenic lipoprotein exposure. From a nutritional standpoint, this makes non-HDL cholesterol particularly sensitive to diet-related metabolic stress. Diets high in ultra-processed foods, added sugars, and saturated fats are associated with elevations in non-HDL cholesterol, whereas improvements in diet quality may contribute to favorable changes in non-HDL cholesterol. Therefore, non-HDL cholesterol may have utility not only as a screening marker but also as a potential indicator for monitoring dietary interventions in adolescents; however, this application remains largely theoretical and warrants further investigation.

These characteristics align well with real-world public health settings, where fasting blood sampling is often impractical in school- or community-based health examinations. Non-HDL cholesterol circumvents this barrier while retaining strong biological relevance, enabling broader implementation of lipid assessment in youth populations.

From a population health perspective, integrating non-HDL cholesterol into adolescent screening programs may support earlier identification of individuals who could benefit most from lifestyle intervention. This approach shifts the focus from late-stage disease detection to upstream prevention, emphasizing dietary quality, physical activity, and metabolic health. In societies undergoing rapid nutritional transition, such as Korea and many other countries, this strategy may be particularly valuable in mitigating the long-term burden of cardiovascular disease.

Taken together, the close pathophysiological link between diet, postprandial hyperlipidemia, and non-HDL cholesterol strengthens the argument for its use in nutrition-oriented prevention frameworks. By bridging lipid biology with practical public health implementation, non-HDL cholesterol provides a pragmatic tool for translating nutritional science into early, actionable cardiovascular risk reduction among adolescents.

Beyond population-level prevention strategies, an additional consideration is the potential role of gene–environment interactions in shaping adolescent lipid profiles. Emerging evidence suggests that individual responses to dietary exposures are partly genetically determined and that genetic susceptibility may modify the effects of nutrition, obesity, and metabolic stress on atherogenic lipoprotein metabolism. Thus, similar dietary patterns may result in substantially different lipid phenotypes among individuals. From this perspective, non-HDL cholesterol may serve not only as a population-level screening marker but also as an integrative biomarker reflecting the combined influence of genetic predisposition and environmental exposures. Future studies incorporating genetic information may further refine precision nutrition and personalized cardiovascular prevention strategies in youth [31].

## 6. From Adolescence to Lifelong Cardiovascular Health

Adolescence represents a critical transitional period in the life-course trajectory of cardiovascular health. Although clinical cardiovascular disease rarely manifests during this stage, metabolic and vascular alterations that precede overt disease often begin to accumulate silently. Dyslipidemia in adolescence, particularly when driven by diet-related and metabolic factors, may therefore serve as an early signal of future cardiovascular vulnerability rather than a transient or benign abnormality.

Emerging evidence suggests that lipid abnormalities in youth track into adulthood, contributing to the development of subclinical atherosclerosis and adverse cardiac remodeling later in life [19]. Non-HDL cholesterol, by reflecting the total burden of atherogenic lipoproteins, may be especially informative in this regard [32]. Unlike isolated fasting LDL cholesterol measurements, non-HDL cholesterol captures sustained exposure to remnant lipoproteins and postprandial lipid stress, which are increasingly recognized as contributors to vascular dysfunction and myocardial impairment over time [33].

This perspective is particularly relevant to the growing burden of heart failure with preserved ejection fraction (HFpEF), a condition closely linked to obesity, insulin resistance, and chronic low-grade inflammation. HFpEF is increasingly recognized as a systemic disorder arising from cumulative cardiometabolic stress rather than solely from traditional ischemic pathways. Although longitudinal studies have demonstrated that adverse lipid profiles in youth are associated with future cardiovascular disease and subclinical atherosclerosis [34,35,36], whether adolescent non-HDL cholesterol is directly associated with the subsequent development of heart failure phenotypes, including HFpEF, remains unknown. Nevertheless, from a pathophysiological perspective, early-life exposure to adverse lipid environments, including postprandial hyperlipidemia, may contribute to endothelial dysfunction, myocardial stiffening, and microvascular inflammation, which are key features of HFpEF. Therefore, adolescent non-HDL cholesterol may represent a biologically plausible marker of future cardiometabolic risk and potentially of later-life heart failure phenotypes, although this hypothesis requires confirmation in dedicated longitudinal studies.

From a preventive standpoint, identifying adolescents with elevated non-HDL cholesterol provides an opportunity to intervene before irreversible cardiovascular changes occur. Nutritional and lifestyle modifications implemented during adolescence have the potential to alter long-term risk trajectories more effectively than interventions initiated later in adulthood. By serving as a feasible and biologically meaningful screening marker, non-HDL cholesterol enables earlier engagement in preventive strategies that address the root causes of cardiometabolic disease.

Importantly, a life-course approach to cardiovascular prevention emphasizes continuity rather than isolated time points. Non-HDL cholesterol measured in adolescence may inform longitudinal risk assessment, guiding follow-up into adulthood and facilitating timely transitions from lifestyle-focused interventions to more intensive management when necessary. Such an approach supports a shift from reactive treatment to proactive prevention.

In summary, the relevance of non-HDL cholesterol extends beyond adolescent screening alone. By linking early nutritional and metabolic disturbances to lifelong cardiovascular outcomes, non-HDL cholesterol occupies a strategic position within life-course prevention frameworks. Its integration into adolescent health assessments may therefore contribute not only to improved short-term risk detection but also to sustained cardiovascular health across the lifespan.

## 7. Future Directions and Conclusions

While the growing body of evidence supports the use of non-HDL cholesterol as a practical screening marker in adolescents, several important areas warrant further investigation. Longitudinal studies are needed to clarify how adolescent non-HDL cholesterol levels track into adulthood and to determine whether they are associated with long-term cardiovascular outcomes, including subclinical atherosclerosis and future heart failure phenotypes. Such data would strengthen the rationale for incorporating non-HDL cholesterol into routine life-course risk assessment frameworks. Future research should also focus on elucidating the complex interactions among genetic predisposition, dietary exposures, obesity, insulin resistance, and non-HDL cholesterol trajectories across the life course. Integrating genetic and environmental information may help identify individuals who are particularly susceptible to adverse lipid responses and could further advance precision prevention and personalized nutrition strategies.

Intervention studies also represent a critical next step. Although non-HDL cholesterol is closely linked to dietary patterns and metabolic health, prospective trials evaluating the impact of nutrition-focused and lifestyle interventions on non-HDL cholesterol trajectories in adolescents remain limited. Understanding the responsiveness of non-HDL cholesterol to specific dietary modifications—such as reductions in ultra-processed food intake or increases in dietary fiber—would enhance its utility not only as a screening tool but also as a target for preventive strategies.

In addition, population-based studies across diverse ethnic, cultural, and nutritional contexts are necessary to confirm the generalizability of current findings. Dietary habits, food environments, and metabolic risk profiles vary widely between populations, and the performance of non-HDL cholesterol as a screening marker may be influenced by these factors. Comparative studies could help refine threshold values and inform context-specific screening recommendations.

Despite these remaining questions, the available evidence already provides a strong foundation for the pragmatic use of non-HDL cholesterol in adolescent health assessments. The Korean national population-based study adds important real-world support, demonstrating that non-HDL cholesterol reliably identifies dyslipidemia across sexes and independently of family history. When combined with its biological relevance, non-fasting convenience, and close association with nutrition-related metabolic stress, non-HDL cholesterol emerges as a compelling first-line screening marker.

In conclusion, non-HDL cholesterol represents a bridge between lipid biology, nutritional science, and public health implementation. Its integration into adolescent screening programs has the potential to shift cardiovascular prevention upstream, enabling earlier, more equitable, and more effective intervention. Although long-term trial evidence linking adolescent non-HDL cholesterol-guided screening directly to reductions in adult cardiovascular events remains limited, the biological plausibility and consistency of observational data support its use as a pragmatic risk marker in youth. As societies continue to grapple with the long-term consequences of modern dietary patterns, non-HDL cholesterol offers a practical and meaningful tool for promoting lifelong cardiovascular health beginning in adolescence.

## Data Availability

No new data were created or analyzed in this study. Data sharing is not applicable to this article.

## Abbreviations

HFpEF	heart failure with preserved ejection fraction
IDL	intermediate-density lipoprotein
LDL	low-density lipoprotein
non-HD	non-high-density lipoprotein
VLDL	very-low-density lipoprotein
